# PKCδ regulates integrin α_V_β_3_ expression and transformed growth of K-ras dependent lung cancer cells

**DOI:** 10.18632/oncotarget.7560

**Published:** 2016-02-21

**Authors:** Jennifer M. Symonds, Angela M. Ohm, Aik-Choon Tan, Mary E. Reyland

**Affiliations:** ^1^ Program in Cancer Biology, The Graduate School, Aurora, CO, USA; ^2^ The Department of Craniofacial Biology, School of Dental Medicine, Aurora, CO, USA; ^3^ The Department of Medical Oncology, School of Medicine University of Colorado Anschutz Medical Campus, Aurora, CO, USA; ^4^ Matrix and Morphogenesis Section, NIDCR, NIH, Bethesda, MD, USA

**Keywords:** PKCδ, lung cancer, integrins, KRAS, anchorage independent growth

## Abstract

We have previously shown that Protein Kinase C delta (PKCδ) functions as a tumor promoter in non-small cell lung cancer (NSCLC), specifically in the context of K-ras addiction. Here we define a novel PKCδ -> integrin α_V_β_3_->Extracellular signal-Regulated Kinase (ERK) pathway that regulates the transformed growth of K-ras dependent NSCLC cells. To explore how PKCδ regulates tumorigenesis, we performed mRNA expression analysis in four *KRAS* mutant NSCLC cell lines that stably express scrambled shRNA or a PKCδ targeted shRNA. Analysis of PKCδ-dependent mRNA expression identified 3183 regulated genes, 210 of which were specifically regulated in K-ras dependent cells. Genes that regulate extracellular matrix and focal adhesion pathways were most highly represented in this later group. In particular, expression of the integrin pair, α_V_β_3_, was specifically reduced in K-ras dependent cells with depletion of PKCδ, and correlated with reduced ERK activation and reduced transformed growth as assayed by clonogenic survival. Re-expression of PKCδ restored *ITGAV* and *ITGB3* mRNA expression, ERK activation and transformed growth, and this could be blocked by pretreatment with a α_V_β_3_ function-blocking antibody, demonstrating a requirement for integrin α_V_β_3_ downstream of PKCδ. Similarly, expression of integrin α_V_ restored ERK activation and transformed growth in PKCδ depleted cells, and this could also be inhibited by pretreatment with PD98059. Our studies demonstrate an essential role for α_V_β_3_ and ERK signalingdownstream of PKCδ in regulating the survival of K-ras dependent NSCLC cells, and identify PKCδ as a novel therapeutic target for the subset of NSCLC patients with K-ras dependent tumors.

## INTRODUCTION

Lung cancer is the leading cause of cancer-related death in men and women, with the majority of those tumors diagnosed as non-small cell lung cancer (NSCLC) [1, 2]. NSCLC may be further categorized by the driver mutations found in certain subtypes. Oncogenic *KRAS* mutations are found in approximately 25% of adenocarcinomas, the largest sub-type of NSCLC [3]. Tumors harboring oncogenic *KRAS* mutations, regardless of tumor site, have poor clinical outcomes. Recently, several groups have reported that a subset of *KRAS* mutant tumors are fully reliant on the *KRAS* oncogene for their survival, i.e., are K-ras dependent, while others have lost their addiction to K-ras and are presumably dependent on alternative survival pathways [4]. Understanding the signaling pathways that regulate tumorigenesis in these K-ras dependent cancer cells will be important for the development of effective therapies for patients with these treatment refractive tumors.

The PKC family is comprised of 10 serine/threonine kinases that have been implicated in numerous biological processes, including proliferation, the immune response, survival, and apoptosis [5]. PKCε and PKCι/λ are most strongly associated with human cancer, while the function of other isoforms in cancer, including PKCδ, appears to be context dependent [6]. Studies in PKCδ knock-out mice have confirmed a role for this kinase in cell death in response to irradiation [7] and during mammary gland involution [8]. *In vitro*, depletion or inhibition of PKCδ results in resistance to multiple apoptotic stimuli [8-10]. While most non-transformed cells use PKCδ for apoptotic signaling, in many cancer cells these pathways are disabled. This may underlie the somewhat paradoxical observation that in certain oncogenic contexts PKCδ appears to be required for cancer cell growth. For example, studies from our lab have shown that PKCδ is required for tumorigenesis driven by oncogenic K-ras [11] and that PKCδ regulates proliferation of Her2/neu driven tumors *in vivo* and in human breast cancer cells *in vitro* [12]. PKCδ has also been shown to promote tumor progression of human pancreatic cancer, to function as a tumor promoter in a mouse model of skin cancer, and to negatively regulate the proliferation and survival of cancer stem cells [13-15].

To understand the mechanism by which PKCδ functions as a tumor promoter, we analyzed PKCδ regulated genes in K-ras dependent and independent NSCLC cells. Our studies identify focal adhesion signaling and extracellular matrix (ECM) genes as differentially regulated in K-ras dependent versus K-ras independent NSCLC cells. These include the integrin genes, *ITGAV* and *ITGB3* that code for the heterodimer, integrin α_V_β_3_. Increased expression of integrin α_V_β_3_ correlates with a poor prognosis in some human tumors [16]. Integrin α_V_β_3_ acts as a receptor for ECM ligands, including fibronectin and vitronectin, and is a well-established regulator of invasion and anchorage-independent growth *in vitro* [17, 18]. Integrin α_V_β_3_ can also have ligand-independent functions in tumor cells [18] and recent studies show that un-ligated integrin α_V_β_3_ can drive cancer cell stemness and drug resistance through activation of K-ras and RalB [19]. Our studies describe a novel PKCδ->integrin α_V_β_3_-> Extracellular signal-Regulated Kinase (ERK) pathway that is important for regulation of transformed growth specifically in K-ras dependent NSCLC cells, and suggest that perturbation of this pathway may be a novel therapeutic strategy for the subset of NSCLC patients with K-ras dependent tumors.

## RESULTS

### Expression profiling of genes regulated by PKCδ in K-ras mutant NSCLC cells

We have previously shown that PKCδ is required for tumorigenesis driven by oncogenic K-ras and for the survival of human NSCLC cell lines that are dependent on K-ras [11]. To further understand the function of PKCδ in the context of oncogenic K-ras we sought to identify genes and functional pathways whose expression is specifically regulated by PKCδ. Transcriptional profiling using Affymetrix GeneChip human genome arrays was performed in two K-ras dependent (H2009 and H441) and two K-ras independent (A549 and H460) NSCLC cell lines that stably express shRNA targeting either the coding region of PKCδ (δ193) or a scrambled non-targeting sequence (δscr). Using a 1.25 fold cut-off, our analysis revealed 3183 genes that show a statistically significant change in gene expression in all cell lines with depletion of PKCδ regardless of their K-ras dependency status. Analysis of gene expression in H2009 and H441 cells revealed 210 genes significantly regulated in both cell lines; 116 genes were down-regulated and 94 genes were up-regulated with depletion of PKCδ (Table S1). In K-ras independent cells, 124 genes were significantly regulated in both cell lines; 77 genes were down-regulated, while 47 genes were up-regulated with depletion of PKCδ (Table S1). Notably, 23 of the 116 genes that were down regulated in K-ras dependent cells were also down regulated in K-ras independent cells. Common down-regulated genes reflect the diverse biological functions of PKCδ and include *RAB23*, a small GTPase in the Ras superfamily; sorting nexin-27 (*SNX-27*); the metabolic enzymes adenosine deaminase (*ADA*) and galactosylceramidase (*GALC*); diacylglycerol kinase alpha (*DGKA*) which regulates diacylglycerol levels and PKC activation at membranes; the cytokine receptors, *IL6ST* and *LIFR*; and *BNIP3L*, an inducer of apoptosis. No up-regulated genes were shared between K-ras dependent and independent cell lines.

**Figure 1:**
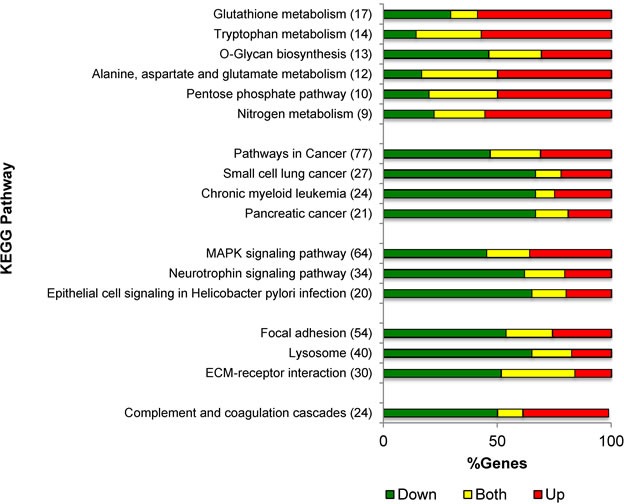
PKCδ regulation of gene expression pathways Distribution of gene expression changes by KEGG pathway for genes regulated by PKCδ across all four cell lines. Shown is the number of genes regulated in each pathway, and the percent of genes with increased expression (red), decreased expression (green) or both increased and decreased expression (yellow) with depletion of PKCδ for each conserved KEGG pathway from Table 1.

To gain insight into the biological pathways controlled by PKCδ in NSCLC, we evaluated the RNA expression profiles of δscr and δ193 expressing NSCLC cells utilizing the KEGG pathway analysis tool within NIH DAVID v6.7 [20-22]. Seventeen KEGG pathways were identified as significantly regulated by PKCδ regardless of K-ras dependency status (Table 1). The top three KEGG pathways identified across all four cells lines were: *Pathways in cancer*, *MAPK signaling*, and *Focal adhesion* (Table 1). In addition, a large number of metabolic pathways were also identified as regulated by PKCδ, including pathways important for glutathione and O-Glycan synthesis, and for amino acid, nitrogen and tryptophan metabolism (Table 1). The percentage of genes whose expression increased or decreased with depletion of PKCδ for each KEGG pathway is shown graphically in Figure 1. For KEGG pathways involved in cancer and cell signaling, the majority of genes showed decreased expression upon depletion of PKCδ. In contrast, the expression of genes in KEGG pathways involved in metabolic processes typically increased upon depletion of PKCδ.

**Table:** KEGG Pathways regulated by PKCδ in K-ras mutant NSCLC cells regardless of K-ras dependency status

KEGG Pathway	Gene number	*p-value*
Pathways in cancer	77	0.0182
MAPK signaling	64	0.0208
Focal Adhesion	54	0.0035
Lysosome	40	0.0001
Neurotrophin signaling	34	0.0164
ECM-receptor interaction	31	0.0003
Small cell lung cancer	27	0.0041
Complement and coagulation cascades	24	0.0024
Chronic myeloid leukemia	24	0.0075
Pancreatic cancer	21	0.0355
Epithelial cell signaling in Helicobacter pylori	20	0.0376
Glutathione metabolism	17	0.0157
Tryptophan metabolism	14	0.0246
O-Glycan biosynthesis	13	0.0051
Alanine, aspartate and glutamate metabolism	12	0.0194
Pentose phosphate	10	0.0302
Nitrogen metabolism	9	0.0489

All statistically significant (*p* < 0.05) KEGG pathways shown.

Six KEGG pathways were significantly enriched in PKCδ depleted K-ras dependent H2009 and H441 cells (Table 2). These include genes whose products regulate ECM-receptor interaction such as *RELN, TNC* and *ZYX* (increased with PKCδ depletion), and *TNXB, ITGAV, ITGA3* and *ITGB3* (decreased with PKCδ depletion), and genes that regulate cell survival and proliferation (*TP53, AKT3, MYC* and *NRAS*). KEGG pathways significantly enriched in K-ras independent cell lines A549 and H460 are also listed in Table 2. The majority of genes in these pathways encode components of receptor signaling pathways and their ligands including *ERBB2, JAK1, IL20RB, TLR3* and *LIFR*. Of note, the KEGG pathway *Chronic myeloid leukemia*, is enriched in both K-ras dependent and independent NSCLC cells, however, with the exception of *NRAS*, the specific genes regulated in this pathway are unique for each subtype.

**Table:** KEGG Pathways regulated by PKCδ in K-ras dependent and independent NSCLC cell lines

KEGG Pathway	Gene number	Genes	*p*-value
**K-ras Dependent**			
Focal adhesion	9	Up: AKT3, RELN, TNC, ZYX	0.006
		Down: ITGAV, ITGA3, ITGB3, MET, TNXB	
ECM-receptor interaction	8	Up: RELN, TNC,	0.0001
		Down: AGRN, DAG1, ITGAV, ITGA3, ITGB3, TNXB	
Chronic myeloid leukemia	5	Up: AKT3, CBLB, MYC	0.019
		Down: NRAS, TP53	
Small cell lung cancer	5	Up: AKT3, MYC	0.028
		Down: ITGAV, ITGA3, TP53	
Sphingolipid metabolism	4	Up: -	0.016
		Down: ASAH1, GALC, NEU1, SGMS2	
Endometrial cancer	4	Up: AKT3, MYC	0.034
		Down: NRAS, TP53	
**K-ras Independent**			
Pathways in cancer	7	Up: FOS, MDM2	0.041
		Down: CDK6, ERBB2, JAK1, NFKBIA, NRAS	
Prostate cancer	5	Up: CREB3, MDM2	0.005
		Down: ERBB2, NFKBIA, NRAS	
Jak-STAT signaling	5	Up: IL20RB, SPRY4	0.032
		Down: IL6ST, JAK1, LIFR	
Chronic myeloid leukemia	4	Up: MDM2	0.021
		Down: CDK6, NFKBIA, NRAS	
Toll-like receptor signaling	4	Up: FOS,MAP2K3	0.044
		Down: NFKBIA, TLR3	
Bladder cancer	3	Up: MDM2	0.043
		Down: ERBB2, NRAS	

All significant (p < 0.05) KEGG pathways are shown for 210 genes regulated by PKCδ in K-ras dependent (H2009 and H441 cells); 94 genes up regulated and 116 genes down regulated. All significant (p < 0.05) KEGG pathways are shown for 124 genes regulated by PKCδ in K-ras independent (A549 and H460 cells), 47 genes up regulated and 77 genes down regulated.

### PKCδ regulates integrin gene expression in K-ras dependent NSCLC cells

**Figure 2:**
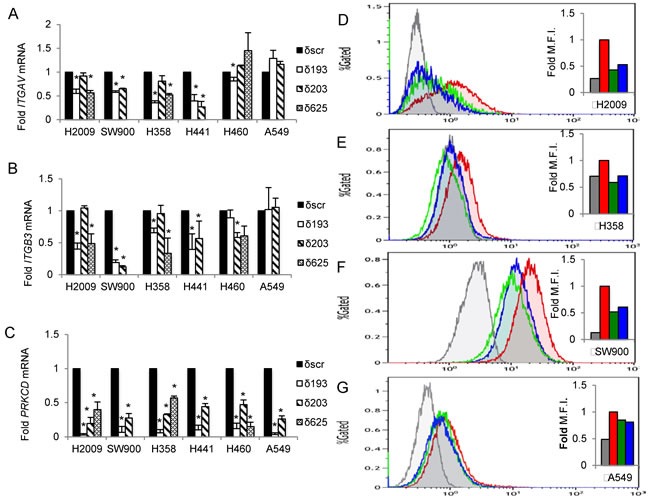
PKCδ regulates *ITGAV* and *ITGB3* mRNA expression in K-ras dependent NSCLC cell lines PKCδ was depleted in K-ras dependent cells (H2009, SW900, H358, H441) or K-ras independent cells (H460, A549) using unique shRNAs (δ193, δ203, δ625) or a scrambled control shRNA (δscr) as described in Materials and Methods. **A.**-**C.** qRT-PCR values of *ITGAV* (A), *ITGB3* (B) and *PRKCD* (C) mRNA expressed as fold of δscr control; black bars = δscr, white bars = δ193, diagonal lined bars = δ203, and hashed bars = δ625 shRNA. Error bars are standard error of the mean of three or more experiments, *p < 0.05, Student's *t*-test. **D.**-**G.** NSCLC cells expressing shRNA to PKCδ (δ193, δ203) or δscr were analyzed for integrin α_V_β_3_ expression at the cell surface by flow cytometry with an antibody that recognizes the integrin α_V_β_3_ heterodimer (LM609). K-ras dependent NSCLC cells H2009 (D), H358 (E), and SW900 (F), and K-ras independent cells A549 (G). Inset shows histogram of median fluorescent intensity, red = δscr, green = δ193, blue = δ203, grey = anti-mouse IgG. This experiment was repeated three times; a representative experiment is shown.

Further examination of gene expression changes in the KEGG pathways *Focal adhesion* and *ECM-receptor interaction* reveals enrichment in integrins, ECM proteins, and downstream signaling enzymes. We next validated these genes by qRT-PCR (Table S2). From this data set, we identified integrin genes whose expression was differentially regulated by PKCδ, based on K-ras dependency status. The products of two differentially regulated integrins, *ITGAV* and *ITGB3,* form the α_V_β_3_ heterodimer, an integrin pair previously shown to regulate tumor cell survival and tumor metastasis in a ligand-independent fashion [17]. We confirmed the differential expression of *ITGAV* and *ITGB3* mRNA using a panel of four K-ras dependent NSCLC cell lines (H2009, SW900, H358 and H441) and two K-ras independent cell lines (A549 and H460). Up to three unique shRNAs were used to deplete PKCδ, including δ193 and δ203 that target the coding region, and δ625 that targets the 3′ UTR of the PKCδ mRNA (Figure 2C). Analysis of *ITGAV* mRNA showed a significant decrease in K-ras dependent NSCLC cells with PKCδ depletion, while no consistent change was observed in A549 or H460 K-ras independent NSCLC cells depleted of PKCδ (Figure 2A). Analysis of *ITGB3* mRNA expression revealed a similar pattern (Figure 2B). To determine if changes in mRNA expression correlate with changes in integrin α_V_β_3_ expression at the cell surface, we used an antibody that specifically recognizes the integrin α_V_β_3_ dimer. Consistent with mRNA expression, depletion of PKCδ led to a decrease in cell surface integrin α_V_β_3_ in K-ras dependent H2009, H358 and SW900 cells, relative to cells expressing δscr shRNA (Figure 2D, 2E and 2F). Stable depletion of PKCδ in the K-ras independent cell line A549 also resulted in a slight but consistent decrease in cell surface expression of integrin α_V_β_3_ (Figure 2G) even though no change in mRNA expression was observed (Figure 2A and 2B), suggesting that PKCδ may also contribute to mobilization of integrin α_V_β_3_ to the cell surface.

**Figure 3:**
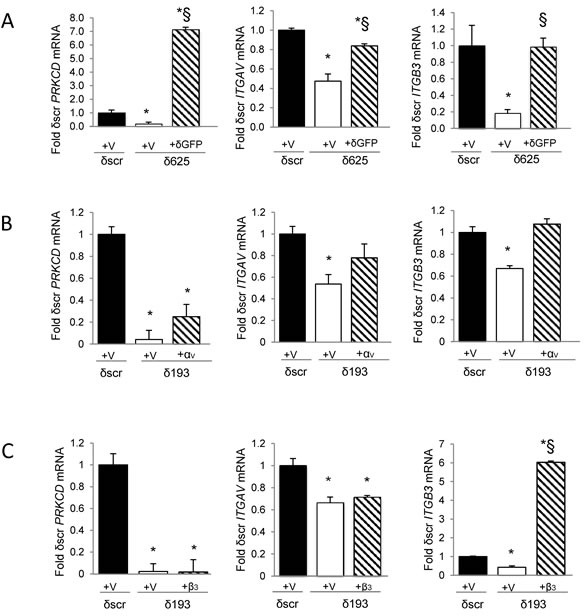
Rescue of PKCδ increases expression of *ITGAV* and *ITGB3* mRNA **A.** H2009 δscr or δ625 cells were transduced with Ad-GFP (V) or Ad-GFP-PKCδ (δGFP). **B.** H2009 δscr or δ193 cells were transiently transfected with pLenti-siLuc (V) or pLenti-*ITGAV* (α_V_). **C.** H2009 δscr or δ193 cells were transiently transfected with pBABE (V) or pBABE-*ITGB3* (β_3_). *PRKCD* (left), *ITGAV* (middle), or *ITGB3* (right) mRNA was assayed as described in Materials and Methods and is expressed as fold of δscr control. For panels A, B and C, black bars = δscr + indicated vector, white bars = δ625 (A) or δ193 (B, C) + indicated vector, and diagonal lined bars = δ625 + δGFP (A), δ193 + α_V_ (B), or δ193 + β_3_ (C). Values shown are an average of three experiments; error bars are standard error of the mean; *p < 0.05 compared to δscr; § *p* < 0.05 compared to δ193 or δ625 (Student's *t*-test).

To confirm that PKCδ regulates *ITGAV* and *ITGB3* gene expression, we rescued PKCδ expression in H2009 δ625 cells by adenoviral transduction of GFP-tagged PKCδ (Ad-GFP-PKCδ) or Ad-GFP (Figure 3A). Re-expression of PKCδ was verified by qRT-PCR (Figure 3A, left). Rescue of PKCδ resulted in increased expression of both *ITGAV* and *ITGB3* mRNA (Figure 3A, middle and right). To determine if expression of *ITGAV* and *ITGB3* are coordinately regulated, we restored integrin α_V_ expression by transient transfection of pLenti-*ITGAV* and assayed *ITGB3* mRNA. Surprisingly, expression of *ITGAV* cDNA restored expression of both *ITGAV* and *ITGB3* mRNA expression (Figure 3B, middle and right). This suggests that reduced expression of *ITGB3* in PKCδ depleted cells may be a consequence of reduced expression of *ITGAV* mRNA. However, re-expression of integrin α_V_ also increased *PRKCD* mRNA (Figure 3B, left) and protein expression (Figure 6E), which could contribute to the increase in *ITGB3* mRNA expression observed. In contrast, expression of *ITGB3* cDNA (Figure 3C, right) had no effect on *ITGAV* or *PRKCD* mRNA levels (Figure 3C, left and middle).

### PKCδ regulates transformed growth of K-ras independent NSCLC cells

**Figure 4:**
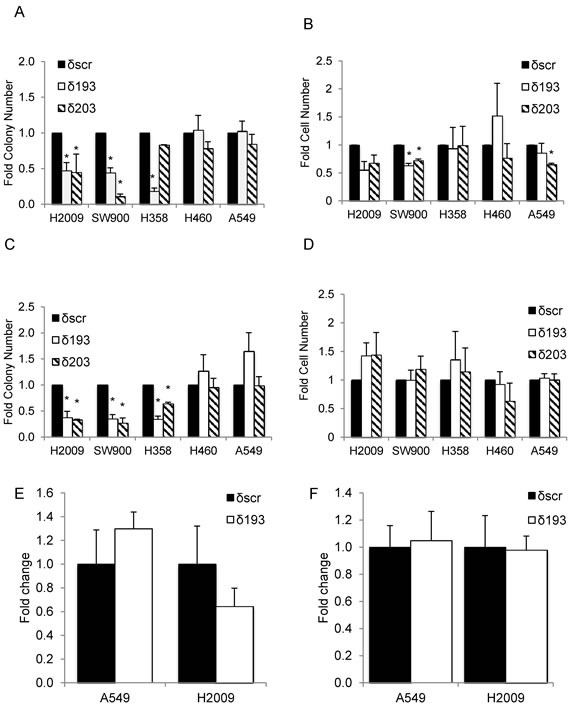
PKCδ regulates survival in K-ras dependent cell lines K-ras dependent (H2009, SW900, H358) or K-ras independent (H460, A549) δscr, δ193 or δ203 cells were cultured on plastic (adherent culture, panels **A.**, **B.** and **E.**) or poly-HEMA coated plates (suspension culture, panels **C.**, **D.** and **F.**). Panels A and C show clonogenic survival; panels B and D show number of viable cells after 24 hours of culture as assayed by Trypan Blue exclusion. Black bars = δscr, white bars = δ193, diagonal lined bars = δ203 shRNA expressing cells. For panels E and F, apoptotic cells in adherent (E) or suspension (F) cultures were assayed using a Yo-Pro assay as described in Materials and Methods. Data is shown as fold of δscr control; black bars = δscr, white bars = δ193. Values shown are an average of three experiments, error bars are standard error of the mean, *p < 0.05 as compared to δscr.

Integrin α_V_β_3_ expression is associated with metastasis in human tumors, presumably through its ability to transmit survival signals in anchorage-independent environments [18]. To determine if decreased expression of integrin α_V_β_3_ upon depletion of PKCδ correlates with reduced transformed growth, we assayed the colony forming ability of NSCLC cells that were detached and re-plated immediately into a clonogenic survival assay (“adherent culture”, Figure 4A), and of cells that were cultured for 24 hours on poly-HEMA coated plates to prevent adhesion, and then re-plated into a clonogenic survival assay (“suspension culture”, Figure 4C). Clonogenic growth under both conditions was significantly reduced with PKCδ depletion in three K-ras dependent NSCLC cell lines (H2009, SW900, and H358), while two K-ras independent cell lines (H460 and A549) showed no decrease with PKCδ depletion. The differences in clonal survival observed between these subpopulations of NSCLC cells were not the result of decreased cell viability (Figure 4B and 4D). Similarly, no significant difference in apoptosis was detected between δscr and δ193 cells under either culture condition (Figure 4E and 4F). Thus, it is likely that the reduced transformed growth observed in K-ras dependent cells with depletion of PKCδ reflects a diminished ability to establish colonies under clonogenic culture conditions.

**Figure 5:**
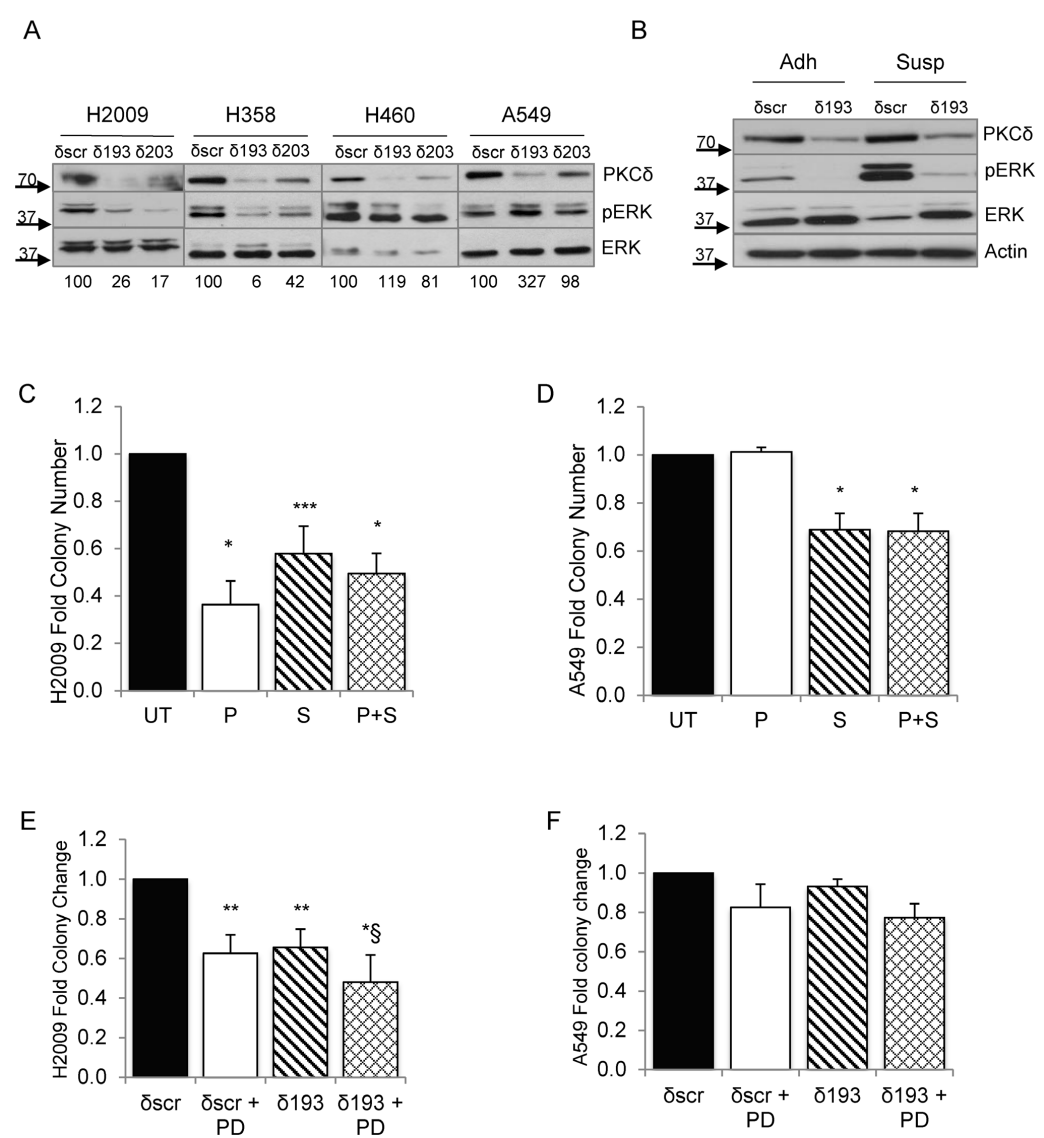
ERK activation is regulated PKCδ and integrin α_V_ **A.** A549, H460, H2009, and H358 δscr, δ193 or δ203 cells were cultured on poly-HEMA coated plates for 24 hrs. Cells were harvested and probed for expression of the indicated proteins by immunoblot; values under blots show fold pERK/ERK for each cell line as determined by densitometry. **B.** H2009 δscr and δ193 cells were cultured under adherent conditions (Adh) or on poly-HEMA coated plates (Susp) for 4 hrs prior to harvest. Cells were harvested and probed for expression of the indicated proteins by immunoblot. For panels **C.** and **D.**, H2009 or A549 cells were maintained in suspension for 24 hours prior to plating for a clonogenic assay. Cells were left untreated (“UT”, black bars) or treated with 60 uM PD90859 as follows: during plating only (“P”, white bars), during suspension culture only (“S”, diagonal lined bars), or during plating and suspension culture (“P+S”, hashed bars). For panels **E.** and **F.**, H2009 δscr or δ193 cells (E), or A549 δscr or δ193 cells (F) were maintained in suspension culture with or without the addition of 60 uM PD98059 for 24 hours prior to plating for a clonogenic assay. For both panels, black bars = δscr, white bars = δscr + PD98059, diagonal lined bars = δ193, and hashed lines = δ193 + PD98059. Values shown are an average of three experiments; error bars are standard error of the mean. *p < 0.05 compared to δSCR, **p < 0.08 compared to δSCR, ***p < 0.10, § *p* < 0.1 compared to δ193, Student's *t*-test.

As both PKCδ and integrin α_V_β_3_ can function as upstream activators of ERK [11], a pathway essential for survival when cells lose their attachment to the ECM [23], we explored the contribution of ERK activation to the transformed growth of K-ras mutant NSCLC cells. As shown in Figure 5A, following suspension culture for 24 hours, ERK activation was lower in H2009 and H358 cells depleted of PKCδ (δ193 and δ203) compared to δscr cells, while neither A549 δ193 and δ203 or H460 δ193 cells showed reduced ERK activation. In some experiments a slight decrease in ERK activation was seen in H460 δ203 cells, however this reduction was much less than that seen in either H2009 or H358 δ193 or δ203 cells. The value under the blot indicates the ratio of pERK/ERK for each cell line (Figure 5A). Activation of ERK in adherent H2009 δ193 cells, and following suspension for 4 hour, was also reduced compared to H2009 δscr cells (Figure 5B). To address the contribution of ERK activity to clonogenic survival of K-ras mutant NSCLC cells, we pre-treated A549 or H2009 cells with the MEK inhibitor, PD98059, either during plating and clonogenic growth (Figure 5C and 5D, “P”), during suspension prior to plating (Figure 5C and 5D, “S”), or during both phases (Figure 5C and 5D, “P+S”). H2009 cells showed a dramatic decrease in clonogenic survival with ERK inhibition regardless of when PD98059 was included (Figure 5C). For A549 cells inclusion of PD98059 also inhibited colony formation, albeit to a lesser extent, and only when included during suspension (Figure 5D). Treatment of H2009 δscr and δ193 cells with PD98059 during suspension and plating also inhibited clonogenic growth (Figure 5E), however PD98059 had no significant effect on the clonogenic growth of A549 δscr or δ193 cells (Figure 5F).

### PKCδ regulates the transformed growth of K-ras dependent NSCLC cells through an integrin α_V_β_3_ and ERK dependent pathway

**Figure 6:**
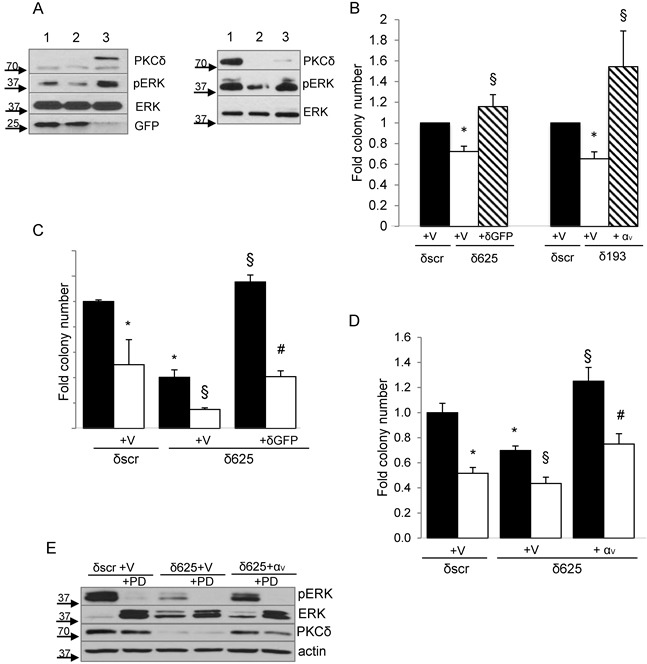
PKCδ regulation of transformed growth requires integrin α_V_β_3_ **A.**, Left panel, H2009 δscr cells were transduced with Ad-GFP (lane 1), or H2009 δ625 cells were transduced with Ad-GFP (lane 2) or Ad-GFP-PKCδ (lane 3). Right panel, H2009-δscr cells were transiently transfected with pLenti-siLuc (lane 1) or H2009 δ193 cells were transiently transfected with pLenti-siLuc (lane 2) or pLenti-ITGAV (lane 3). Cells were harvested and probed for expression of the indicated proteins by immunoblot. Arrows indicate the position of endogenous and GFP- PKCδ. **B.** For PKCδ reconstitution, H2009 δscr (black bars) and δ625 cells (white bars) were transduced with Ad-GFP (V), or δ625 cells were transduced with Ad-GFP-PKCδ (δGFP) (diagonal lined bars) prior to plating in the clonogenic assay. For integrin α_V_ rescue, H2009 δscr (black bars) and δ193 cells (white bars) were transiently transfected with pLenti-siLuc (V), or H2009 δ193 were transiently transfected pLenti-*ITGAV* (αv) (diagonal lined bars) prior to plating in the clonogenic assay. Values shown are an average of six experiments. Error bars represent the standard error of the mean. **C.** H2009 δscr or δ625 cells (black bars) were transduced with Ad-GFP (V) or Ad-GFP-PKCδ (δGFP) and put in suspension culture for 24 hours with the inclusion of 20 ug/ml of an integrin α_V_β_3_ function blocking antibody (LM609) (white bars) or 20ug/ml anti-mouse IgG (black bars) prior to plating in a clonogenic assay. This experiment was repeated four times; a representative experiment is shown. Error bars represent the standard error of the mean. **D.** H2009 δscr or δ625 cells (black bars) were transfected with pLenti-siLuc (V) or pLenti-*ITGAV* (αv) as indicated, and then put in suspension culture for 24 hours without (black bars) or with (white bars) the inclusion of 60uM PD98059 prior to plating in a clonogenic assay. Sixty uM PD98059 was also included in the plating media. This experiment was repeated three times; a representative experiment is shown. Error bars represent the standard error of the mean. For graphs B, C, D: *p < 0.05 compared to δscr, § *p* < 0.05 compared to δ193 or δ625, # *p* < 0.05 compared to δ625-δGFP/αv. **E**. A representative immunoblot of cells transfected in (D) and probed for expression of the indicated proteins.

To determine if reduced ERK signaling contributes to the suppression of transformed growth observed with PKCδ depletion, we asked if reconstitution of PKCδ could restore ERK activation in H2009 PKCδ depleted cells. As expected, ERK activation was decreased in H2009 δ625 cells (Figure 6A, left, lane 2) compared to δscr cells (Figure 6A, left, lane 1), however this was completely rescued in cells transduced with Ad-GFP-PKCδ (Figure 6A left, lane 2 versus lane 3). Similarly, expression of integrin α_V_ also restored ERK activation in H2009 PKCδ depleted cells (Figure 6A, right, lane 2 versus lane 3). Reconstitution of either PKCδ or integrin α_V_ expression likewise completely restored transformed growth (Figure 6B). To ask if integrin α_V_β_3_ is required for PKCδ regulation of transformed growth, H2009 δ625 cells were transduced with Ad-GFP-PKCδ or Ad-GFP and transformed growth was assayed in the presence of an integrin α_V_β_3_ blocking antibody. Inclusion of the integrin α_V_β_3_ blocking antibody completely blocked the increase in transformed growth seen with re-expression of PKCδ, indicating an absolute requirement for α_V_β_3_ signaling downstream of PKCδ (Figure 6C). As our data shows that both PKCδ and integrin α_V_β_3_ regulate ERK activation and transformed growth of K-ras dependent NSCLC cells (Figure 6A and 6B), and that integrin α_V_β_3_ is a downstream effector of PKCδ in this pathway, we next asked if integrin α_V_β_3_ regulation of ERK activation is required for transformed growth. H2009 δ625 cells were transfected with pLenti-*ITGAV* or a pLenti-siLuc control vector and PD98059 was included during the clonogenic survival assay. As shown in Figure 6D, expression of *ITGAV* increased transformed growth of H2009 δ625 cells, and this could be nearly completely blocked by PD98059, indicating a requirement for ERK downstream of both PKCδ and integrin α_V_β_3_ for survival under anchorage independent conditions. Taken together, our data defines a novel mechanism through which PKCδ regulation of α_V_β_3_ expression and ERK activation controls transformed growth of NSCLC cells in the context of K-ras dependency. Of note, our studies also show that *ITGAV* can regulate PKCδ mRNA and protein expression (Figure 3B and 6E), thus transformed growth in this context may be sustained in part through a positive feedback mechanism that assures activation of ERK dependent survival pathways.

## DISCUSSION

Oncogenic mutations in *KRAS* are found in about 25% of lung adenocarcinomas, however many tumors with mutations in *KRAS* are no longer “dependent” on K-ras for survival, thus targeting these tumors will depend on understanding the molecular underpinnings of K-ras dependence [3, 4]. Our previous studies have defined PKCδ as a key mediator of K-ras dependent tumorigenesis in NSCLC [11]. In an effort to understand how PKCδ functions in the context of oncogenic *KRAS,* we analyzed PKCδ regulated gene expression in K-ras dependent and independent NSCLC cell lines. Our studies identify the integrin heterodimer, α_V_β_3_, as a critical target of PKCδ in the context of K-ras dependency, and define a novel PKCδ -> integrin α_V_β_3_-> ERK pathway that regulates the transformed growth of human K-ras dependent NSCLC cells.

Expression profiling of genes regulated by PKCδ reveals changes in the expression of over 3000 genes in *KRAS* mutated NSCLC cells, confirming earlier studies by Caino, *et al*. that defined PKCδ as a major transcriptional regulator of genes induced by phorbol ester [24]. Similar to Caino *et al*., we found that the majority of these genes were down regulated with depletion of PKCδ, supporting a role for PKCδ in positively regulating their transcription. KEGG pathway analysis suggests that PKCδ can influence a wide variety of cellular pathways and processes, a concept supported by functional data from many labs [25, 26] (Table 1). Two main themes are evident from our analysis. First, PKCδ likely contributes to the regulation of metabolic processes that control nutrient availability and oxidative stress, as depletion of PKCδ increases the expression of genes that drive amino acid, nitrogen and glutathione metabolism. Genes in the pentose phosphate pathway, important for production of NADPH, are also regulated by PKCδ, consistent with known roles for PKCδ in regulation of ROS and NADPH [27, 28]. Second, PKCδ is important for supporting survival signaling, including “outside-in” signaling. This is consistent with the pro-survival properties attributed to PKCδ in tumor models, including known roles for PKCδ in invasion and migration [11, 29-31], and our own data that shows increased PKCδ expression correlates with a worse prognosis in breast cancer patients [6, 12]. Notably, several genes in the *ECM-receptor interaction* and *Focal adhesion* KEGG pathways identified in our studies were also identified as PKCδ regulated genes by Caino *et al.* including *TNC, ZYX, MET* and *TNXB* [24].

We show that pro-survival integrin α_V_β_3_ is a target of PKCδ in K-ras dependent NSCLC cells, and that re-expression of PKCδ restores expression of *ITGAV* and *ITGB3* mRNA (Figure 3). Our studies suggest that expression of the integrin α_V_β_3_ heterodimer is coordinated at the level of transcription or mRNA processing/stability. While others have shown that increased expression of one integrin subunit can stimulate cell surface expression of its binding partner, transcriptional regulation by partner integrins has not previously been reported [32, 33]. Previous reports do however suggest coordinated transcriptional regulation of *ITGAV and ITGB3 genes*, including a recent study that demonstrates inhibition of *ITGAV* and *ITGB3* transcription by Myc in a breast cancer model [33]. Likewise, thyroid hormone has been shown to regulate transcription of *ITGAV and ITGB3* through an ERK dependent process [34]. In our studies re-expression of integrin α_V_ also increases *PRKCD* mRNA and protein expression, thus we cannot rule out a role for PKCδ in regulation of *ITGB3* mRNA expression.

Our findings identify PKCδ as a potential regulator of tumor progression and metastasis through modulation of integrin α_V_β_3_regulated survival pathways. Notably, PKC isoforms, including PKCδ, have been previously shown to regulate integrin adhesion [29], signaling [35-37], and α_V_β_3-_integrin mediated invasion [38]. PKCδ has also been linked to survival signaling through regulation of growth factor receptor and receptor tyrosine kinase activation of MAPK family of signaling cascades [7, 11, 12, 39-43]. Activation of ERK is required for survival of cells in suspension [23], and alterations in ERK activation may contribute to anoikis resistance, a hallmark of metastatic tumor cells [44]. We provide evidence of ERK regulation by PKCδ in K-ras dependent, but not K-ras independent NSCLC cells, which could account for the specific effect of PKCδ depletion on clonogenic survival (Figure 5A). As depletion of PKCδ does not reduce survival in suspension culture or induce apoptosis, ERK activation downstream of PKCδ during suspension may be required to prime cells for survival under clonogenic conditions. PKCδ regulates ERK activation through integrin α_V_β_3_, as re-expression of either PKCδ or integrin α_V_ restores transformed growth and activates ERK (Figure 6A and 6B). Furthermore, we show that restoration of transformed growth in H2009 δ625 cells reconstituted with PKCδ requires integrin α_V_β_3_ (Figure 6C). Our studies however do not exclude a role for PKCδ in regulation of ERK activation upstream of integrin α_V_β_3,_ or through integrin α_V_β_3_ independent mechanisms. In this regard, recent studies by Kurihara *et al*. demonstrate a PKC-ERK-α_V_β_3_ pathway that regulates Tumor Necrosis Factor-α production in monocytes [45].

Patients harboring tumors with oncogenic *KRAS* mutations are generally refractive to available therapies, resulting in poor clinical outcomes. Our studies support exploration of PKCδ as a drug target in KRAS mutant lung cancer. We show that PKCδ regulation of integrin α_V_β_3_ survival signaling is specific for K-ras dependent NSCLC cells. As K-ras dependent and independent phenotypes have been demonstrated in other human cancers with oncogenic K-ras, including pancreatic adenocarcinoma and colon cancer [4], the pathways defined here may contribute to tumor progression in many types of human cancer. Based on previous studies by the Cheresh lab, which correlate increased integrin α_V_β_3_ expression with tumor progression, the PKCδ -> integrin α_V_β_3_-> ERK pathway we describe may have consequences for the metastatic potential of cancer cells [18]. PKCδ has been implicated as a pro-metastatic factor in breast cancer models [46] and in many tumor phenotypes associated with metastasis [47] such as migration and invasion [11]. Our studies suggest that inhibition of PKCδ in K-ras dependent NSCLC may decrease the expression of integrin α_V_β_3_, a known regulator of cell survival *in vitro*, and a factor associated with metastasis *in vivo* and in patients. Identifying patients most likely to benefit from targeting the PKCδ survival pathway will depend on genetic and/or functional markers of PKCδ dependency. The co-segregation of PKCδ and K-ras dependency will help to focus this effort, while providing new therapeutic options for patients with K-ras dependent cancers.

## MATERIALS AND METHODS

### NSCLC cell lines and PKCδ depletion

NSCLC cell lines were acquired through the University of Colorado Denver Lung Cancer SPORE cell bank. Cell line profiling for authentication was done at the DNA sequencing Core at University of Colorado Anschutz Medical Campus using the ABI profiler plus and ABI Identifiler profiling kits. NCI-H2009 (H2009), NCI-H358 (H358), SW-900 (SW900), NCI-H441 (H441), NCI-H460 (H460) and A549 cells were cultured in RPMI-1640 with 2 mM L-glutamine and supplemented with 10% fetal bovine serum. Stable depletion of PKCδ was done as previously described using lentiviral constructs containing shRNA to human PKCδ [(pLKO-TRC00010193 (δ193) or pLKO-TRC00010203 (δ203) from Open Biosystems, or TRCN0000196625 (δ625) from SigmaAldrich] or a scrambled shRNA control (pLKO-scrambled (δscr) from Open Biosystems) [11]. Stable cell lines were continuously maintained in 2 μg/mL puromycin. For PKCδ rescue, H2009 δ625 or δscr cells were transduced with an adenovirus that expresses GFP-tagged murine PKCδ (Ad-GFP-PKCδ) or GFP adenovirus (Ad-GFP) at multiplicity of infection (MOI) of 25 or 50. For integrin α_V_ rescue, H2009 δ193 or δscr cells were transfected with pLenti-*ITGAV* or pLenti-siLUC (1.5 ng DNA/5 × 10^4^ cells). For integrin β3 rescue H2009 δ193 or δscr cells were transfected with pBabe-*ITGB3* or control pBABE using Fugene (Promega #E2311) as transfection reagent. In some experiments an integrin α_V_β_3_ function blocking antibody (LM609) (Millipore #MAB1976) or anti-mouse IgG (Millipore #CBL610) was included at 20 ug/ml.

### DNA microarray and data analysis

mRNA from three biological replicates of NSCLC cells stably transduced with δscr or δ193 shRNA were profiled on Affymetrix Human Gene 1.0 ST gene arrays by the Microarray Core, University of Colorado Denver Anschutz Medical Campus. Raw data were normalized using Affymetrix Power Tools (APT) using Robust Multiarray Average method. Genes whose expression were statistically significantly different in the three δ193 replicates, (Student's *t*-test, *p* < 0.05), and which showed a 1.25 fold change or greater over the δscr replicates, were selected for further analysis. Candidate genes were analyzed for enriched pathways using National Institutes of Health Database for Annotation, Visualization, and Integrated Discovery (NIH DAVID) program [48, 49] and the KEGG analysis program [21, 22]. Raw microarray data has been deposited to NCBI Gene Expression Omnibus with the accession number GSE72788.

### Quantitative real time polymerase chain reaction (qRT-PCR)

Analysis was completed as previously described [50]. In brief, total RNA was purified from cells using RNeasy mini kits (Qiagen) and were reverse transcribed using random hexamers and Moloney murine leukemia virus reverse transcriptase (Thermo #EP0741). The reverse transcription reactions were analyzed by PCR using Absolute Blue qPCR SYBR Green Mix (Thermo #AB-4166) in an iCycler (BioRad) thermal cycler. qRT-PCR analysis of mRNA was carried out with forward and reverse primers as indicated (Supplementary data, Table S3). Expression was normalized to glyceraldehyde-3-phosphate dehydrogenase (GAPDH) mRNA expression as measured by qRT-PCR in replicate samples. Data are presented as “relative expression,” or “fold δscr mRNA.”

### Clonogenic survival assay and suspension culture

Tissue culture plates were coated with 1% poly (2-hydroxyethyl methacrylate) (polyHEMA; Sigma P3932) solution in 95% ethanol, and allowed to completely dry at room temperature. Cells (1 × 10^5^ cells/mL) were cultured for 24 hours on 1% polyHEMA plates to prevent adhesion, collected, washed with PBS and 2 mM EDTA and replated into 6-well plates (100 - 1000 cells/well). For some experiments ERK signaling was inhibited by pre-treating NSCLC cell lines with 60 μM PD98059 for 45 minutes prior to plating. In some cases cells were maintained in PD98059 for the duration of the clonogenic assay. Colonies were stained with crystal violet (0.5% crystal violet, 6% glutaraldehyde), and quantified using ImageJ [20, 51].

### Flow cytometry

Cells were cultured under normal adherent conditions for 48 hours prior to analysis, then lifted with Ethylenediaminetetraacetic acid (EDTA) and washed three times in phosphate-buffered saline (PBS). Non-specific binding was blocked with goat serum, and cells were incubated with primary antibody to integrin α_V_β_3_ (LM609) (Millipore #MAB1976) or anti-mouse IgG, negative control (Millipore #CBL610). Cells were washed in PBS with 0.5% bovine serum albumin and 0.1% sodium azide and incubated with phycoerythrin-conjugated secondary antibody (BD#550589). Median fluorescence was measured by the University of Colorado Anschutz Medical Campus Flow Cytometry Core on Gallios flow cytometry machine and analyzed on Kaluza (Beckman Coulter). To assay apoptosis, A549 or H2009 δscr or δ193 cells were plated at a concentration of 1 × 10^5 cells/ml on either 1% polyhema coated plates (suspended) cells or on regular tissue culture plates (attached) for 24 hours. Cells were harvested and stained for flow cytometry using TrypLE express dissociation reagent containing a mixture of 0.1 μM Yo-Pro and 8.1 μM Hoechst 33342 stain (Chromatin Condensation/Membrane Permeability/Dead Cell Apoptosis Kit with Hoechst 33342/YO-PRO®-1 and PI for Flow Cytometry, Invitrogen, Catalog #V23201). Cells were then washed with PBS and analyzed by flow cytometry as described above.

### Immunoblot analysis

Immunoblotting was performed as previously described [7]. Antibodies to phosphorylated ERK1/2 (#9101) and ERK1/2 (#4695) were purchased from Cell Signaling Technologies. The anti-PKCδ antibody was purchased from Santa Cruz (sc-937); anti-actin-HRP and anti-GFP were purchased from Abcam (ab49900 and ab290, respectively).

## SUPPLEMENTARY MATERIAL TABLES



## References

[1] Siegel RL, Miller KD, Jemal A (2015). Cancer statistics, 2015. CA.

[2] Kenfield SA, Wei EK, Stampfer MJ, Rosner BA, Colditz GA (2008). Comparison of aspects of smoking among the four histological types of lung cancer. Tobacco Control.

[3] Beau-Faller M, Legrain M, Voegeli A-C, Guérin E, Lavaux T, Ruppert A-M (2009). Detection of K-Ras mutations in tumour samples of patients with non-small cell lung cancer using PNA-mediated PCR clamping. Br J Cancer.

[4] Singh A, Greninger P, Rhodes D, Koopman L, Violette S, Bardeesy N (2009). A gene expression signature associated with ‘K-Ras addiction’ reveals regulators of EMT and tumor cell survival. Cancer Cell.

[5] Newton AC (2009). Lipid activation of protein kinases. The Journal of Lipid Research.

[6] Garg R, Benedetti LG, Abera MB, Wang H, Abba MC, Kazanietz MG (2014). Protein kinase C and cancer: what we know and what we do not. Oncogene.

[7] Humphries MJ, Limesand KH, Schneider JC, Nakayama KI, Anderson SM, Reyland ME (2006). Suppression of apoptosis in the protein kinase Cdelta null mouse in vivo. Journal of Biological Chemistry.

[8] Allen-Petersen BL, Miller MR, Neville MC, Anderson SM, Nakayama KI, Reyland ME (2010). Loss of protein kinase C delta alters mammary gland development and apoptosis. Cell Death Dis.

[9] Humphries MJ, Ohm AM, Schaack J, Adwan TS, Reyland ME (2007). Tyrosine phosphorylation regulates nuclear translocation of PKCδ. Oncogene.

[10] Leitges M, Mayr M, Braun U, Mayr U, Li C, Pfister G (2001). Exacerbated vein graft arteriosclerosis in protein kinase Cdelta-null mice. J Clin Invest.

[11] Symonds JM, Ohm AM, Carter CJ, Heasley LE, Boyle TA, Franklin WA (2011). Protein kinase C δ is a downstream effector of oncogenic K-ras in lung tumors. Cancer Research.

[12] Allen-Petersen BL, Carter CJ, Ohm AM, Reyland ME (2014). Protein kinase Cδ is required for ErbB2-driven mammary gland tumorigenesis and negatively correlates with prognosis in human breast cancer. Oncogene.

[13] Reddig PJ, Dreckschmidt NE, Ahrens H, Simsiman R, Tseng CP, Zou J (1999). Transgenic mice overexpressing protein kinase Cdelta in the epidermis are resistant to skin tumor promotion by 12-O-tetradecanoylphorbol-13-acetate. Cancer Research.

[14] Chen Z, Forman LW, Williams RM, Faller DV (2014). Protein kinase C-δ inactivation inhibits the proliferation and survival of cancer stem cells in culture and in vivo. BMC Cancer.

[15] Mauro LV, Grossoni VC, Urtreger AJ, Yang C, Colombo LL, Morandi A (2010). PKC Delta (PKCδ) Promotes Tumoral Progression of Human Ductal Pancreatic Cancer. Pancreas.

[16] Desgrosellier JS, Cheresh DA (2010). Integrins in cancer: biological implications and therapeutic opportunities. Nat Rev Cancer.

[17] van der Flier A, Sonnenberg A (2001). Function and interactions of integrins. Cell and Tissue Research.

[18] Desgrosellier JS, Barnes LA, Shields DJ, Huang M, Lau SK, Prévost N (2009). An integrin αvβ3-c-Src oncogenic unit promotes anchorage-independence and tumor progression. Nat Med.

[19] Seguin L, Kato S, Franovic A, Camargo MF, Lesperance J, Elliott KC (2014). An integrin β3-KRAS-RalB complex drives tumour stemness and resistance to EGFR inhibition. Nat Cell Biol.

[20] Schneider CA, Rasband WS, Eliceiri KW (2012). NIH Image to ImageJ: 25 years of image analysis. Nature Methods.

[21] Kanehisa M, Goto S, Sato Y, Kawashima M, Furumichi M, Tanabe M (2014). Data, information, knowledge and principle: back to metabolism in KEGG. Nucleic Acids Research.

[22] Kanehisa M, Goto S (1999). KEGG: Kyoto Encyclopedia of Genes and Genomes. Nucleic Acids Research.

[23] Collins NL, Reginato MJ, Paulus JK, Sgroi DC, Labaer J, Brugge JS (2005). G1/S cell cycle arrest provides anoikis resistance through Erk-mediated Bim suppression. Molecular and Cellular Biology.

[24] Caino MC, Burstin von VA, Lopez-Haber C, Kazanietz MG (2011). Differential regulation of gene expression by protein kinase C isozymes as determined by genome-wide expression analysis. J Biol Chem.

[25] Garg R, Caino MC, Kazanietz MG (2013). Regulation of Transcriptional Networks by PKC Isozymes: Identification of c-Rel as a Key Transcription Factor for PKC-Regulated Genes. PLoS ONE.

[26] Reyland ME (2009). Protein kinase C isoforms: Multi-functional regulators of cell life and death. Front Biosci (Landmark Ed).

[27] Fontayne A, Dang PM-C, Gougerot-Pocidalo M-A, Benna El J (2002). Phosphorylation of p47 phoxSites by PKC α, βΙΙ, δ, and ζ: Effect on Binding to p22 phoxand on NADPH Oxidase Activation. Biochemistry.

[28] Brown GE, Stewart MQ, Liu H, Ha V-L, Yaffe MB (2003). A Novel Assay System Implicates PtdIns(3,4)P2, PtdIns(3)P, and PKCδ in Intracellular Production of Reactive Oxygen Species by the NADPH Oxidase. Molecular Cell.

[29] Chae YC, Kim KL, Ha SH, Kim J, Suh P-G, Ryu SH (2010). Protein kinase Cdelta-mediated phosphorylation of phospholipase D controls integrin-mediated cell spreading. Molecular and Cellular Biology.

[30] Sarkar S, Yong VW (2010). Reduction of protein kinase C delta attenuates tenascin-C stimulated glioma invasion in three-dimensional matrix. Carcinogenesis.

[31] Kho DH, Bae JA, Lee JH, Cho HJ, Cho SH, Seo Y-W (2009). KITENIN recruits Dishevelled/PKC delta to form a functional complex and controls the migration and invasiveness of colorectal cancer cells. Gut.

[32] Kim LT, Yamada KM (1997). The regulation of expression of integrin receptors. Proc Soc Exp Biol Med.

[33] Liu H, Radisky DC, Yang D, Xu R, Radisky ES, Bissell MJ (2012). MYC suppresses cancer metastasis by direct transcriptional silencing of αv and β3 integrin subunits. Nat Cell Biol.

[34] Shinderman-Maman E, Cohen K, Weingarten C, Nabriski D, Twito O, Baraf L (2015). The thyroid hormone-αvβ3 integrin axis in ovarian cancer: regulation of gene transcription and MAPK-dependent proliferation. Oncogene.

[35] Alt A, Gartsbein M, Ohba M, Kuroki T, Tennenbaum T (2004). Differential regulation of α6β4 integrin by PKC isoforms in murine skin keratinocytes. Biochemical and Biophysical Research Communications.

[36] Carduner L, Picot CR, Leroy-Dudal J, Blay L, Kellouche S, Carreiras F (2014). Cell cycle arrest or survival signaling through αv integrins, activation of PKC and ERK1/2 lead to anoikis resistance of ovarian cancer spheroids. Experimental Cell Research.

[37] Rucci N, DiGiacinto C, Orrù L, Millimaggi D, Baron R, Teti A (2005). A novel protein kinase C alpha-dependent signal to ERK1/2 activated by alphaVbeta3 integrin in osteoclasts and in Chinese hamster ovary (CHO) cells. Journal of Cell Science.

[38] Putnam AJ, Schulz VV, Freiter EM, Bill HM, Miranti CK (2009). Src, PKCalpha, and PKCdelta are required for alphavbeta3 integrin-mediated metastatic melanoma invasion. Cell Commun Signal.

[39] Quadros MR, Connelly S, Kari C (2006). Research Paper EGFR-Dependent Downregulation of Bim in Epithelial Cells Requires MAPK and PKC-δ Activities. Cancer Biology & Therapy.

[40] Xia S, Chen Z, Forman LW, Faller DV (2009). PKCδ survival signaling in cells containing an activated p21Ras protein requires PDK1. Cellular Signalling.

[41] Kharait S, Dhir R, Lauffenburger D, Wells A (2006). Protein kinase Cδ signaling downstream of the EGF receptor mediates migration and invasiveness of prostate cancer cells. Biochemical and Biophysical Research Communications.

[42] Iwabu A, Smith K, Allen FD, Lauffenburger DA, Wells A (2004). Epidermal growth factor induces fibroblast contractility and motility via a protein kinase C delta-dependent pathway. Journal of Biological Chemistry.

[43] Paugh BS, Paugh SW, Bryan L, Kapitonov D, Wilczynska KM, Gopalan SM (2008). EGF regulates plasminogen activator inhibitor-1 (PAI-1) by a pathway involving c-Src, PKCdelta, and sphingosine kinase 1 in glioblastoma cells. FASEB J.

[44] Guadamillas MC, Cerezo A, Del Pozo MA (2011). Overcoming anoikis—pathways to anchorage-independent growth in cancer. Journal of Cell Science.

[45] Kurihara Y, Nakahara T, Furue M (2011). αVβ3-integrin expression through ERK activation mediates cell attachment and is necessary for production of tumor necrosis factor alpha in monocytic THP-1 cells stimulated by phorbol myristate acetate. Cell Immunol.

[46] Kiley SC, Clark KJ, Goodnough M, Welch DR, Jaken S (1999). Protein Kinase C δ Involvement in Mammary Tumor Cell Metastasis. Cancer Research.

[47] Hanahan D, Weinberg RA (2011). Hallmarks of cancer: the next generation. Cell.

[48] Huang DW, Sherman BT, Lempicki RA (2008). Systematic and integrative analysis of large gene lists using DAVID bioinformatics resources. Nat Protoc.

[49] Huang DW, Sherman BT, Lempicki RA (2009). Bioinformatics enrichment tools: paths toward the comprehensive functional analysis of large gene lists. Nucleic Acids Research.

[50] Kono SA, Marshall ME, Ware KE, Heasley LE (2009). The fibroblast growth factor receptor signaling pathway as a mediator of intrinsic resistance to EGFR-specific tyrosine kinase inhibitors in non-small cell lung cancer. Drug Resistance Updates.

[51] Franken NAP, Rodermond HM, Stap J, Haveman J, van Bree C (2006). Clonogenic assay of cells in vitro. Nat Protoc.

