# Supplementary material for: PKCδ regulates integrin αVβ3 expression and transformed growth of K-ras dependent lung cancer cells
Source: Oncotarget. 2016 Feb 21;7(14):17905–19. doi: 10.18632/oncotarget.7560 (PMC4951259; doi:10.18632/oncotarget.7560)
Supplement: Supplementary file 1 [file oncotarget-07-17905-s001.pdf]

PKCδ regulates integrin $\alpha_V\beta_3$ expression and transformed growth of K-ras dependent lung cancer cells

Supplementary Material

Table S1: Shared genes regulated by PKCδ in K-ras dependent or independent NSCLC cells. Data is the average change in expression of significant genes in both K-ras dependent (H2009 and H441) or K-ras independent (A549 and H460) cell lines (p<0.05). Only genes that were increased or decreased in both cell lines are included for each K-ras subpopulation.

K-ras Dependent				K-ras Independent			
Down (fold change)		Up (fold change)		Down (fold change)		Up (fold change)	
PRKCD	-4.11	CD274	2.61	PRKCD	-3.05	OR51B4	2.34
PI3	-3.58	CALB2	2.54	GPRC5B	-2.24	HBE1	2.24
SPINK6	-3.45	TRPA1	2.49	ADA	-2.09	STC1	2.03
ALDH1A1	-2.72	GFPT2	2.01	RAB23	-1.97	BDKRB1	2.01
RAB23	-2.42	LCP1	1.97	HOXA2	-1.83	CYP1B1	1.99
C9orf5	-2.38	SLC1A3	1.86	CP	-1.82	SLC16A6	1.99
PON2	-2.03	DYNLRB1	1.84	FERMT2	-1.77	TFF1	1.92
CFL1	-2.02	HSD17B10	1.84	TMEM47	-1.72	EDIL3	1.86
GALC	-2.01	MUC13	1.83	C9orf5	-1.70	HHIPL2	1.80
ADA	-1.95	TIMP3	1.79	HOXA3	-1.67	SMOC1	1.76
CALU	-1.93	HOXB9	1.77	TERF2IP	-1.63	TRPA1	1.74
RBM9	-1.91	NR0B1	1.74	RNF157	-1.62	HSD17B10	1.69
SAMD9	-1.87	RELN	1.70	FAM171A1	-1.61	DYNLRB1	1.67
STK38	-1.85	CLU	1.65	SCARA3	-1.59	SLC4A4	1.65
FSTL1|MIR198	-1.82	TMED4	1.64	SNX27	-1.58	GLRX	1.65
CROT	-1.78	BMP6	1.64	LIFR	-1.56	SYTL3	1.61
TERF2IP	-1.77	WARS	1.62	NRP2	-1.56	SCARNA7	1.60

VAMP7	-1.74	GBP1|GBP3	1.61	DGKA	-1.52	MDM2	1.57
ASCC3	-1.74	PMP22	1.61	RBM9	-1.51	ELL2	1.56
LIFR	-1.73	FERMT3	1.59	CTSH	-1.49	IGFBP3	1.55
ITGA3	-1.72	NID2	1.58	CDK6	-1.47	MT1X	1.54
C1orf85	-1.71	B3GALNT2	1.56	NDRG2	-1.46	MCTP1	1.50
SNX27	-1.71	TNC	1.54	RNF185	-1.46	DKFZp686O24166	1.50
TSPAN1	-1.71	HBA1|HBA2	1.52	HMOX1	-1.46	KATNAL1	1.45
HIRA	-1.65	GLP2R	1.52	EDA2R	-1.45	NF2	1.45
NRAS|CSDE1	-1.65	HBA2|HBA1	1.51	PSME3	-1.44	TMED4	1.44
GPRC5A	-1.64	GPT2	1.51	FBLN1	-1.43	SPRY4	1.44
NEK7	-1.64	CENPV	1.50	TULP3	-1.42	CCDC68	1.41
ELF4	-1.64	ANKRD36B	1.49	C5orf25	-1.42	B3GALNT2	1.41
ELF3	-1.64	RPIA	1.49	STRA6	-1.42	SCARNA9L	1.41
AMIGO2	-1.60	MYC	1.48	MRC2	-1.42	ATP2B1	1.39
IL6ST	-1.59	C9orf37	1.48	PON2	-1.40	SLC25A32|DCAF13	1.37
PSME3	-1.59	MTHFD2	1.48	UGP2	-1.40	KRT17	1.35
SMAD5	-1.58	KIAA1804	1.46	TLR3	-1.39	SLC9A2	1.35
RAB5B	-1.57	KCNG1	1.46	JAK1	-1.39	CLGN	1.34
DHRS3	-1.56	LYRM1	1.46	PDK3	-1.39	FOS	1.33
TNFRSF10D	-1.56	COX10	1.45	PLK2	-1.39	SS18	1.33
TM9SF1	-1.55	ADD2	1.44	ARRB1	-1.38	IL20RB	1.33
RBP4	-1.55	PRKD1	1.44	ANKRD54	-1.38	GBA2|CREB3	1.32
GOLIM4	-1.54	NPM3	1.44	PQLC3	-1.38	MAP2K3	1.31
BNIP3L	-1.53	FAM27E3	1.44	SMAD5	-1.38	ZC3HAV1L	1.31
WDR45L	-1.53	MAFG	1.44	BNIP3L	-1.37	IRS2	1.31
YIPF6	-1.53	FAM27E3	1.43	ATP6V1C1	-1.37	CABYR	1.28

FERMT2	-1.53	CUTC	1.42	TMEM139	-1.37	FAM83A	1.28
LCOR	-1.52	TYW3|CRYZ	1.42	CLMN	-1.37	FURIN	1.27
PODXL	-1.51	CABYR	1.42	PLAC8	-1.37	PYGB	1.27
SEPX1	-1.51	PDP2	1.42	ZNF281	-1.36	PLIN3	1.27
F11R|TSTD1	-1.50	NEIL3	1.40	PBK	-1.36		
ASAH1	-1.50	DOT1L	1.40	ZDHHC13	-1.35		
AGRN	-1.50	SLC43A1	1.40	PLA2R1	-1.35		
FYTTD1	-1.50	CREB5	1.39	TSPAN6	-1.35		
NRIP1	-1.49	SPRYD3	1.39	TSPAN33	-1.35		
SFT2D2	-1.49	CPVL	1.39	RNF170	-1.34		
ICAM2	-1.49	PALLD	1.39	C1S	-1.34		
LOC554202	-1.47	UPP1	1.39	PTBP2	-1.34		
EPGN	-1.47	RAP1GAP2	1.38	C6orf72	-1.33		
DGKA	-1.47	CBLB	1.38	IRF2	-1.33		
ITGAV	-1.47	SH3PXD2B	1.38	TM2D2	-1.33		
DAG1	-1.46	PCID2	1.37	C3	-1.32		
ID2	-1.45	SDCCAG3	1.37	AADAC	-1.32		
CCNYL1	-1.45	C17orf75	1.37	ERBB2	-1.32		
SLC39A11	-1.45	MTIF2	1.36	GALC	-1.32		
C6orf72	-1.45	AKT3	1.36	FIG4	-1.32		
FUCA1	-1.45	IL32	1.35	CIB2	-1.32		
NRP2	-1.44	CNRIP1	1.34	NFKBIA	-1.31		
SGMS2	-1.43	MED24	1.34	GLIS3	-1.31		
C4orf34	-1.43	CRLF3	1.34	NRAS|CSDE1	-1.31		
FAM171A1	-1.42	SHISA3	1.33	CNTNAP1	-1.31		
TMBIM4|LOC100133322	-1.42	ZYX	1.33	ARL15	-1.30		

MET	-1.42	CENPH	1.33	SCML2	-1.30		
TAF10	-1.41	NCRNA0015 2	1.32	SEPX1	-1.29		
TP53	-1.41	DPH5	1.32	DIRC2	-1.29		
C20orf3	-1.41	RSL24D1	1.31	IL6ST	-1.29		
ITGB3	-1.41	MPZ	1.31	CSF1	-1.28		
ATXN1	-1.41	SLC16A3	1.31	SEMA4B	-1.27		
ATF6B|TNXB	-1.40	SLC16A9	1.31	NAB1	-1.26		
HOXA2	-1.40	METTL3	1.31	LHFP	-1.26		
ATF6B|TNXB	-1.40	TMEM120B	1.31				
ENTPD7	-1.40	THOC6	1.31				
SYT14	-1.39	POLR3D	1.31				
TMEM63B	-1.38	DNMT3B	1.31				
UBE3B	-1.38	JUND	1.30				
REEP3	-1.38	AKAP12	1.30				
RNF185	-1.36	DTD1	1.30				
FUT8	-1.36	SNRNP27	1.29				
HOXA3	-1.35	STAC	1.29				
EAF1	-1.35	ATG4D|KRI1	1.28				
ATP6V1C1	-1.35	OSBPL1A	1.28				
SLC9A7	-1.35	FKBP11	1.28				
RNF141	-1.34	MRPS23	1.28				
ANPEP	-1.34	SMAP2	1.27				
KIAA1370	-1.34	RPUSD3	1.26				
TMEM56	-1.34	MRPL47	1.26				
DCTN5	-1.34	ZBTB11	1.26				
GPATCH8	-1.34						

GGCX	-1.34							
MYLIP	-1.33							
SSR1	-1.33							
LRRC16A	-1.33							
LTBP3	-1.33							
PPIC	-1.32							
SLC35B4	-1.32							
MAP3K7	-1.32							
PIGQ	-1.31							
MST1R	-1.30							
SLC9A6	-1.30							
YIPF3	-1.30							
SEMA3C	-1.29							
EXOC6B	-1.29							
TMCO3	-1.29							
TMEM9B	-1.29							
DNAJC3	-1.28							
NEU1	-1.27							
C10orf118|MIR2110	-1.26							
HS2ST1	-1.26							
MYD88	-1.26							

Table S2: Validation of Focal adhesion and ECM-receptor interaction gene expression in K-ras mutant NSCLC cells. Gene expression was validated by qRT-PCR in cell lines expressing δ193 and δ203 and compared to expression in cells expressing δscr (see Materials and Methods). Values represent fold change relative to δscr cells. nc= no change with PKCδ depletion; nc*****=predicted down by microarray; nc******=predicted up by microarray.

	**K-ras dependent**		**K-ras independent**	
**Gene**	**H2009**	**H441**	**H460**	**A549**
CFL1	-1.48	-1.97	-1.44	-1.33
COL5A2	1.48	nc*	nc	nc
CPA4	-1.80	-42.49	-3.21	-1.58
DAG	-1.33	-2.29	nc*	-1.43
DUSP5	nc**	5.06	1.28	nc
FN1	nc	-2.30	nc	1.79
ITGA2	-2.42	-2.69	1.28	-1.93
ITGA5	nc	nc	1.74	1.50
ITGA6	nc	-1.73	nc	nc
ITGAV	-1.81	-2.53	-1.33	nc
ITGB1	nc	nc*	nc	nc
ITGB3	-2.78	-2.78	nc	nc
ITGB4	nc	-2.85	1.20	1.91
JUND	1.76	nc*	2.03	nc
LAMA1	1.55	nc	-1.58	nc
LAMA4	-1.87	-3.32	-3.85	7.78
LAMB2	-1.48	-2.88	-1.86	-1.39
LAMC1	nc	nc	nc	nc
LAMC2	-2.04	-2.28	nc	-2.07
LXN	nc	-56.94	nc*	nc
MMP16	1.73	nc	-1.56	nc
MMP19	-	-1.93	-1.80	nc
MMP2	1.12	-2.19	-1.58	2.92
RELN	1.32	nc**	nc	nc
TNC	1.69	1.23	11.79	1.46
TNXB	-2.72	-2.03	2.53	nc
VTN	-1.62	-6.37	nc	nc

Table S3: Primers used for qRT-PCR.

Gene	Forward (5' to 3')	Reverse (5' to 3')
CD274	TGCCGACTACAAGCGAATTACTG	CTGCTTGTCCAGATGACTTCGG
CFL1	GCAACCTATGAGACCAAGGAGAG	TCTTGATGGCGTCCTTGGAGCT
COL5A2	GAAGCCTCCCAGAACATCACTTAC	TTGCCCCTTTGAGAACCACAGC
CPA4	CAATGAAGGGCAAGAACGGAGC	GGTCAGGAAAGTCTGCGGCAAT
DAG	CCACAGTGGTTGGCATTCCTGA	CCAAGATGGCAAAGCCTCCTTC
DUSP5	CAGCATCCTGATTTGAACCCTG	TCCAGAAAAGCCAACATCTTGC
FN1	TGTCTATGCTCAGAATCCAAGCG	CCACATCAGTGAATGCCAGTCC
GAPDH	GCCAAATATGATGACATCAAGAAGG	GGTGTCGCTGTTGAAGTCAGAG
ITGA2	TGTTACCTGCTGGTTGAAAGACG	TGATGCGAAAGTCCCGTTCC
ITGA5	CTCCTTCTTCGGATTCTCAGTGG	TGGTATTAGCCTTGGGTGCTCC
ITGA6	CGAGGTTATGGAACAGCACATTTC	TTTCGGCAGCAGCAGTCACATC
ITGAV	AGGAGAAGGTGCCTACGAAGCT	GCACAGGAAAGTCTTGCTAAGGC
ITGB1	GCCCTCCAGATGACATAGAAAATCC	TGCTGTTCCTTTGCTACGGTTG
ITGB3	AAGCAGAGTGTGTCACGGAACC	CCTCCAGCCAATCTTTTCATCAC
ITGB4	CTCGCCAAGCACAACATCATC	CAGTGAGGAGACAGGGAAATAGGTG
JUND	ATCGACATGGACACGCAGGAGC	CTCCGTGTTCTGACTCTTGAGG
LAMA1	TGGCAGGGAAAAGGTGTGACAG	TCGCAGGTATTCTGAGTGTGTGGG
LAMA4	TGGCTCACCAATCAGTGCTCAG	TCCACATCTCTATCCACCCTGG
LAMB2	ACGGCACTGGGATGATGTAGTC	AGCACACGATAGATGACCTCGC
LAMC1	TGTTCCCAAGACAAAGGAGGTG	CCAAAGTAGCCATCATCACAGAGC
LAMC2	CTGGGAAACTGGAAAGAAGAAGC	TTTAGCAAGATTGGCACGGG
LTX	GCGACTTTTCCGCCTCGGCA	TGGGTAGTTGGTCGGCGGGA
MAP3K7	CAGAGCAACTCTGCCACCAGTA	CATTTGTGGCAGGAACTTGCTCC
MET	TGCACAGTTGGTCCTGCCATGA	CAGCCATAGGACCGTATTTCGG
MMP16	GCAGCACAAGCACATCACTTACAG	CATCAAAGGCACGGCGAATAG
MMP19	CAGTGCCCACAGAACCCAGTC	TGAAAGCATAGGTCTTCCCACG
MMP2	GTGTCTTCCCCTTCACTTTCCTG	ACCACATCTTTCCGTCACTGCG
MYC	GCTCGCCCTCCTACGTTGCG	TGGTCGGCCGTGGAGAAGCT
RELN	GTCTACCTTCCACTCTCCACCA	GTCCAGCATCACAAATCCCTCG
TNC	ATGTCCTCCTGACAGCCGAGAA	AGTCACGGTGAGGTTTTCCAGC
TNXB	CTGGAATCCGACCACAGATACC	TGGGAGGTTCTGAAGGCTTCTC
VTN	TGGACGAAAAGGCAGTGAGG	TGACAGTTGATGCGGGTGAAGG
